# Current Diabetes Self-Management and the Number of Documented Complications in Adults with Type 2 Diabetes: A Cross-Sectional Study

**DOI:** 10.3390/medicina62091777

**Published:** 2026-09-16

**Authors:** Paula-Alexandra Popovici, Andreea Diana Igna, Bianca-Lăcrimioara Petca, Timea Claudia Ghitea, Mihaela Simona Popoviciu

**Affiliations:** 1 Doctoral School of Biological and Biomedical Sciences, University of Oradea, 410087 Oradea, Romania; cipleu.paulaalexandra@student.uoradea.ro (P.-A.P.); igna.andreeadiana@student.uoradea.ro (A.D.I.); criste.biancalacrimioara@student.uoradea.ro (B.-L.P.); 2 Pharmacy Department, Faculty of Medicine and Pharmacy, University of Oradea, 410087 Oradea, Romania; 3 Department of Preclinical Disciplines, Faculty of Medicine and Pharmacy, University of Oradea, 410087 Oradea, Romania; mihaela.popoviciu@didactic.uoradea.ro

**Keywords:** type 2 diabetes, self-management, multimorbidity, outpatient care, cross-sectional studies

## Abstract

*Background and Objectives:* Current self-management behavior may not reflect the long-term exposures that led to established complications in people with type 2 diabetes (T2D). To assess whether Diabetes Self-Management Questionnaire (DSMQ) scores were associated with the number of documented diabetes-related and cardiovascular conditions and whether the finding was robust to alternative outcome constructions. *Materials and Methods:* This single-center cross-sectional reanalysis included 262 adults with T2D. The DSMQ was re-calculated from item-level responses. The primary outcome was an unweighted count of seven database-recorded conditions. Spearman correlation and multivariable Poisson regression with HC3 robust standard errors were used. Sensitivity analyses excluded heart failure, separated microvascular and cardiovascular counts, and substituted estimated glomerular filtration rate <60 mL/min/1.73 m^2^ for the recorded chronic kidney disease field. *Results:* Median DSMQ Sum Scale was 5.10 (IQR 3.13–7.08), and median condition count was 3 (IQR 2–3). No evidence of an unadjusted association was identified (ρ = −0.056; *p* = 0.368). In the adjusted model (*n* = 260), the DSMQ estimate was IRR 0.988 per point (95% CI 0.962–1.014; *p* = 0.355). The result remained nonsignificant after excluding heart failure (IRR 0.993; 95% CI 0.960–1.027), for the microvascular count (IRR 0.993; 95% CI 0.964–1.024), and for the cardiovascular count including heart failure (IRR 0.980; 95% CI 0.945–1.015). Pearson dispersion was 0.587, providing no evidence of overdispersion. *Conclusions:* No evidence of an association between current DSMQ-measured self-management and the number of recorded conditions was identified in this cohort. The result does not demonstrate equivalence and does not imply that sustained self-management is unrelated to long-term complication risk. In practice, contemporary DSMQ scores should guide cur-rent education and support needs, not be used as a proxy for historical com-plication exposure.

## 1. Introduction

Type 2 diabetes (T2D) is a chronic metabolic disease frequently accompanied by microvascular and macrovascular complications. When several conditions coexist, patients face greater treatment burden, polypharmacy, functional limitations, and fragmented care, while clinicians must coordinate glycemic, cardiovascular, renal, neurological, ophthalmological, and behavioral priorities [1,2,3,4]. The number and pattern of coexisting conditions may therefore affect both the feasibility and the content of diabetes self-management.

Diabetes self-management is multidimensional and includes medication-taking, glucose monitoring, dietary behavior, physical activity, problem-solving, and communication with healthcare professionals. In people with T2D and multimorbidity, these activities may be complicated by treatment burden, functional limitations, diabetes distress, limited health literacy, and fragmented care. Lifestyle-medicine case-manager nurses may support the coordination of education, monitoring, lifestyle interventions, and referrals, while community-based nurse-led programs may improve glycemic control and reinforce sustained self-care behaviors [5,6,7,8]. Diabetes education, patient–provider communication, and health literacy may also influence self-management directly and through their relationship with diabetes distress [8]. These findings support a multidisciplinary interpretation of self-management that extends beyond medication adherence alone.

The 16-item Diabetes Self-Management Questionnaire (DSMQ) evaluates behaviors relevant to glycemic management and yields a Sum Scale and four domains: Glucose Management, Dietary Control, Physical Activity, and Health-Care Use [9]. The instrument primarily reflects relatively recent behavior. By contrast, chronic kidney disease, neuropathy, retinopathy, and cardiovascular disease typically arise from exposures accumulated over many years. A cross-sectional association between a current DSMQ score and prevalent complications may therefore be weak even when sustained self-management is important for long-term risk.

The source database contained binary indicators for several conditions but no validated composite severity index. Accordingly, the outcome is de-scribed conservatively as the number of documented conditions, not as a validated cumulative complication burden score. Equal weighting remains clinically simplistic because the conditions differ in severity, prognosis, diagnostic certainty, and effect on daily functioning.

The primary objective was to evaluate whether current DSMQ-measured self-management was associated with the number of seven documented diabetes-related or cardiovascular conditions. Secondary objectives were to test the robustness of the association after excluding heart failure, to analyze microvascular and cardiovascular counts separately, to assess a laboratory-based renal sensitivity definition, and to document the suitability of the Poisson count model. We hypothesized no direction of effect because the cross-sectional design cannot establish whether self-management preceded or followed the recorded conditions.

## 2. Materials and Methods

### 2.1. Study Design, Setting, and Participants

This was a single-center cross-sectional secondary analysis of a database of adults with T2D evaluated in a specialized diabetes and nutrition outpatient setting in Romania between 1 November 2025 and 30 May 2026. The supplied file contained 263 rows; one final administrative row containing variable labels was excluded. Records with numeric age, complete item-level DSMQ responses, and complete binary condition fields were eligible, resulting in 262 participants. The adjusted models used complete cases for DSMQ, age, the database F/M field, T2D duration, and HbA1c.

The analytic file represented all participant records supplied for the stated study period. Participants were included from the complete analytic database available for the study period. Because a separate screening log was not maintained, the number of patients assessed before database inclusion and the reasons for non-enrollment could not be reconstructed. Accordingly, the sample should be interpreted as a database-derived convenience sample, and no claim of consecutive recruitment is made.

### 2.2. Data Sources and Validation

Demographic, behavioral, anthropometric, laboratory, treatment, education, and clinical-condition variables were extracted directly from the CSV file. Derived variables were recalculated from the corresponding source fields whenever possible. Body mass index (BMI) was recalculated using the recorded weight and height. One value outside the permitted F/M coding (“LM”), one implausible HbA1c value of 61%, and one implausible fasting plasma glucose value of 2293 mg/dL were treated as missing in analyses involving these variables. The original values were retained in the source database, which was not modified.

Automated data validation included frequency and range checks, comparison of stored and recalculated DSMQ scores, recalculation of BMI, and comparison of the recorded chronic kidney disease (CKD) field with estimated glomerular filtration rate (eGFR) at the index assessment. Because the analytic CSV did not contain albuminuria measurements or repeated renal assessments, a single eGFR value < 60 mL/min/1.73 m^2^ was interpreted only as reduced kidney function at the index assessment and not as independent confirmation of CKD [10].

The seven conditions included in the primary outcome were additionally verified against the available source medical records by two clinicians with experience in diabetes care and internal medicine. Diagnostic ascertainment was based on clinical documentation, laboratory findings, imaging results, specialist assessments, and hospital discharge records available at or before the index assessment, according to the criteria presented in Table 1. For CKD, evidence of chronicity and other markers of kidney damage were assessed from the source records when available. Disagreements were resolved by consensus between the two reviewers. Diagnoses that lacked sufficient supporting documentation to meet the prespecified criteria were classified as absent from the corresponding condition count.

### 2.3. Diabetes Self-Management

The DSMQ contains 16 items scored from 0 to 3. Items 5, 7, and 10–16 were reverse-keyed before calculation. The Sum Scale included all 16 items. Domain scores used the original allocation: Glucose Management (items 1, 4, 6, 10, and 12), Dietary Control (items 2, 5, 9, and 13), Physical Activity (items 8, 11, and 15), and Health-Care Use (items 3, 7, and 14). Raw sums were transformed to a 0–10 scale, with higher scores indicating more effective current self-management [9]. Stored derived DSMQ fields were not used because they materially disagreed with the item-level recalculation.

### 2.4. Documented Condition Outcomes

The primary outcome was an unweighted count of seven documented conditions: chronic kidney disease, diabetic peripheral neuropathy, diabetic retinopathy, previous myocardial infarction, stroke, peripheral arterial disease, and heart failure. Each condition that met the prespecified diagnostic and documentation criteria presented in Table 1 contributed one point, producing a count ranging from 0 to 7. Diagnoses that could not be confirmed from the available source records were not classified as present.

### 2.5. Statistical Analysis

Continuous variables were summarized as median and interquartile range (IQR), and categorical variables as number and percentage. The unadjusted association between DSMQ scores and condition counts was assessed using Spearman rank correlation. Comparisons by individual condition used Mann–Whitney U tests, with the Benjamini–Hochberg procedure controlling the false discovery rate across related secondary tests [11].

The primary adjusted analysis used Poisson regression with HC3 heteroskedasticity-consistent standard errors. Prespecified covariates were DSMQ Sum Scale, age, the female database code, T2D duration, and HbA1c. The same covariate set was used for the alternative count outcomes. Model suitability was assessed using Pearson χ^2^/df, deviance/df, observed versus Poisson-predicted zero frequencies, maximum predicted count, and Akaike information criterion (AIC). A negative binomial model was fitted as a sensitivity check; because the estimated overdispersion parameter approached zero and fit did not improve, the robust Poisson model was retained.

No a priori sample-size calculation was performed because this was a secondary analysis of a fixed database. Consequently, nonsignificant results were interpreted using confidence intervals and were not treated as evidence of equivalence or proof of no association. Complete-case analysis was used for adjusted models. Tests were two-sided with α = 0.05. Analyses used Python 3.11.15, pandas 2.2.3, SciPy 1.15.2, and statsmodels 0.14.4. Reporting followed the STROBE statement; the checklist is provided in Table S7 [12].

### 2.6. Ethics

The study was conducted in accordance with the Declaration of Helsinki and was approved by the Ethical Committee of the Faculty of Medicine and Pharmacy, University of Oradea (approval no. 41, 31 October 2025), before the study period began on 1 November 2025. The study protocol and consent procedures were verified against the original institutional records. Written informed consent was obtained from all participants before inclusion.

## 3. Results

### 3.1. Analytic Sample and Data Validation

**Figure 1:**
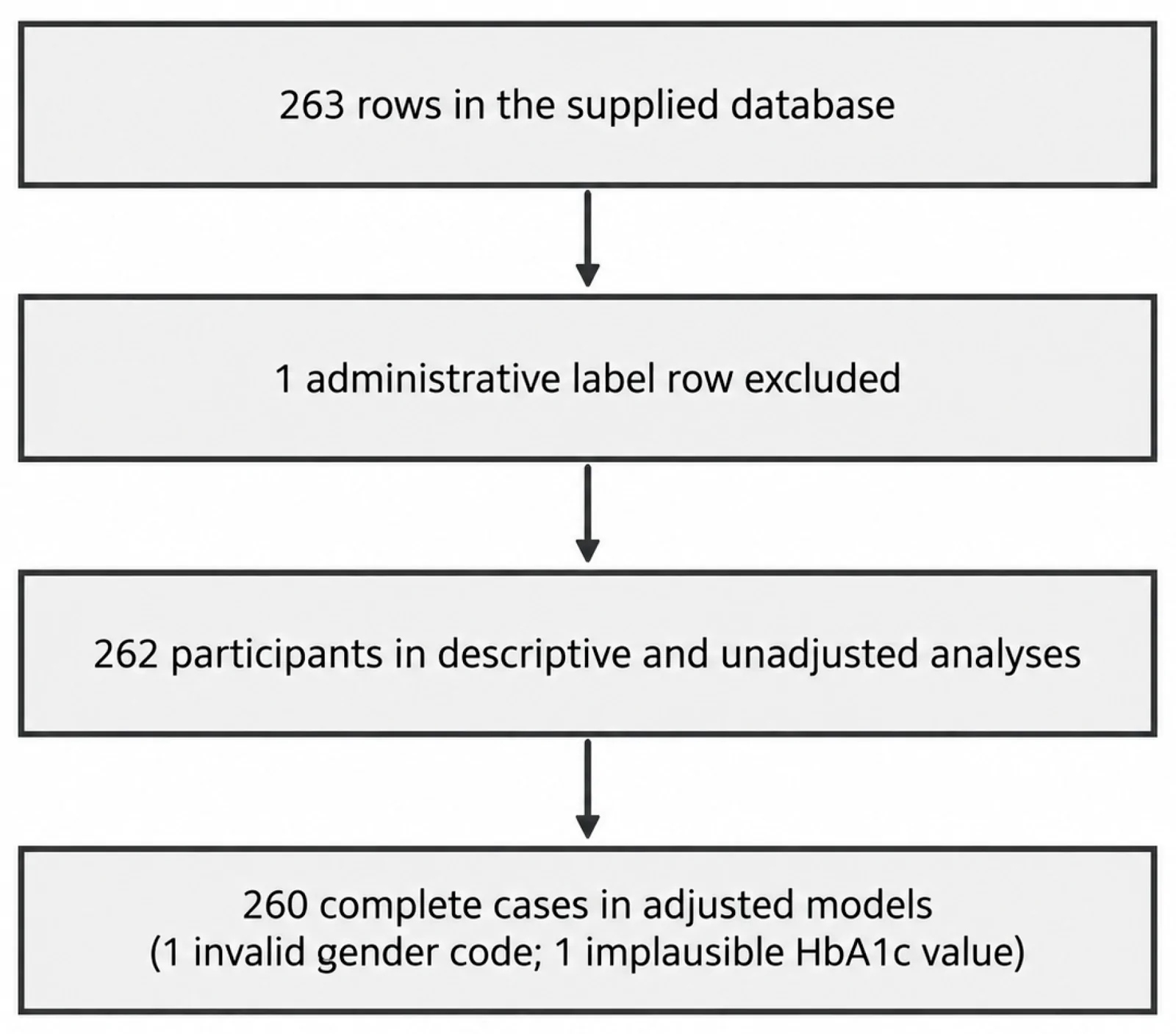
Construction of the analytic sample. HbA1c, glycated hemoglobin.

Of 263 rows in the supplied database, one administrative label row was excluded, leaving 262 participants for descriptive and unadjusted analyses. All 262 had complete item-level DSMQ responses and seven condition fields. Adjusted models included 260 complete cases after exclusion of one invalid database F/M code and one implausible HbA1c value (Figure 1).

Item-level validation showed that 249 of 262 stored DSMQ Sum Scale values (95.0%) differed by more than 0.01 points from the correctly recalculated values. Discrepancies were also present in stored domain fields; recalculated scores were therefore used throughout. Cronbach’s α for the correctly keyed 16 items was 0.916. Recalculated BMI differed from stored BMI by >0.5 kg/m^2^ in 15 participants and by >1.0 kg/m^2^ in nine participants. Source-record review confirmed all condition-positive database entries included in the analysis; therefore, no condition classifications or cumulative counts required modification.

### 3.2. Participant Characteristics and Recorded Conditions

Median age was 64 years (IQR 58–73), 144 of 261 participants with a valid F/M code were female (55.2%), and median T2D duration was 7 years (IQR 4–14). Median recalculated DSMQ Sum Scale was 5.10 (IQR 3.13–7.08), and the median number of documented conditions was 3 (IQR 2–3). Selected characteristics and condition frequencies are shown in Table 2; expanded descriptive results are in Tables S1–S3.

### 3.3. Primary Association and Alternative Outcome Constructions

The DSMQ Sum Scale was not monotonically associated with the seven-condition count (Spearman ρ = −0.056; *p* = 0.368). In the adjusted robust Poisson model, no evidence of an independent association was identified (IRR 0.988 per point; 95% CI 0.962–1.014; *p* = 0.355). The confidence interval indicates that, per one-point higher DSMQ score, the data were compatible with approximately a 3.8% lower to a 1.4% higher expected count. For a five-point score difference, the corresponding IRR was 0.940 (95% CI 0.825–1.071); thus, associations of modest clinical magnitude in either direction cannot be completely excluded.

The DSMQ estimate remained nonsignificant when heart failure was excluded, when microvascular and cardiovascular outcomes were modeled separately, and when the macrovascular count excluded heart failure (Table 3). Recorded CKD was present in 171 participants (65.3%), whereas 74 (28.2%) had eGFR < 60 mL/min/1.73 m^2^ at the index assessment. Ninety-nine participants had recorded CKD with eGFR ≥ 60, and two had eGFR < 60 without recorded CKD; agreement was 61.5%. These findings cannot adjudicate CKD because albuminuria and chronicity were unavailable. Substituting eGFR < 60 for the recorded CKD field did not materially change the DSMQ result (IRR 0.983; 95% CI 0.953–1.014; *p* = 0.290).

### 3.4. Primary Model Covariates and Model Fit

Older age and longer T2D duration were associated with a higher documented-condition count, whereas participants with a female database code had a lower expected count. This finding reflects the coding available in the source database and should not be interpreted as a broader gender-related effect (Table 4). These covariate findings are cross-sectional and descriptive and should not be interpreted causally.

Secondary exploratory analyses are reported in Tables S4–S6. No individual condition or DSMQ domain showed a statistically significant association after false discovery rate correction. Similarly, the exploratory comparison between participants with fewer than four and those with at least four documented conditions did not identify evidence of a difference in DSMQ scores. In the exploratory logistic model, the DSMQ Sum Scale was not associated with the classification of at least four documented conditions (OR 1.055 per point; 95% CI 0.908–1.225; *p* = 0.487).

## 4. Discussion

In this cross-sectional reanalysis, no evidence of an association between current DSMQ-measured self-management and the number of documented conditions was identified. The estimate was close to the null in unadjusted and adjusted analyses and remained nonsignificant after excluding heart failure, separating microvascular and cardiovascular outcomes, excluding heart failure from the macrovascular count, and substituting reduced eGFR for the recorded CKD field. The Poisson model did not show overdispersion, and a negative binomial model did not improve fit.

The central interpretive issue is temporal mismatch. The DSMQ reflects relatively recent self-management, whereas most included conditions develop over years. The present findings therefore do not indicate that sustained self-management is unrelated to the long-term development of complications. Contemporary behavior may also change after diagnosis: intensified education and treatment could improve self-management, whereas pain, visual loss, reduced mobility, fatigue, distress, and treatment burden could impair it. Opposing pathways can attenuate a cross-sectional association [9,13,14,15,16,17,18,19,20,21].

The confidence interval should also temper interpretation. The primary estimate excluded large per-point differences but remained compatible with a 17.5% lower to a 7.1% higher expected count across a five-point DSMQ contrast. Because no a priori sample-size calculation was performed, nonsignificance cannot establish equivalence or rule out smaller associations. Repeated DSMQ measurements and prospectively adjudicated incident outcomes would be required to evaluate whether sustained self-management trajectories predict future complications [11,12,17,22,23,24].

The high frequencies of recorded heart failure, CKD, and peripheral neuropathy require particular caution. Excluding heart failure did not change the DSMQ conclusion, which supports statistical robustness to that component. However, robustness of an association estimate does not validate a diagnosis. The CKD–eGFR comparison illustrates why source-record review is essential: CKD may coexist with eGFR ≥ 60 when other markers of kidney damage are present, while one low eGFR value does not establish chronicity [10,18,19,25,26,27]. Heart failure and peripheral neuropathy were verified using the available specialist and hospital documentation. However, detailed information regarding heart-failure phenotype, ejection fraction, and neuropathy severity was not consistently available. Therefore, these conditions were analyzed as binary documented diagnoses, without differentiation by subtype or severity.

The unweighted count was useful as a transparent summary but is not a clinical severity index. It assigns equal value to heterogeneous conditions, ignores stage and timing, and may combine pathophysiologically related diagnoses. Separate microvascular and cardiovascular models partly address this concern, but future studies should prespecify clinically justified outcome groups and use standardized diagnostic adjudication [28,29,30].

### 4.1. Perspectives for Clinical and Assistive Practice

DSMQ scores and documented complications should be treated as complementary information. A low current DSMQ score can identify behavioral barriers and trigger tailored education even in a person with few complications; conversely, a high score does not imply low historical risk or exclude organ damage. Systematic screening for renal, retinal, neurological, and cardiovascular disease should continue independently of questionnaire results.

For people with multimorbidity, support should be multidisciplinary and responsive to diabetes distress, health literacy, communication needs, physical limitations, social resources, and treatment complexity. Community-based and nurse-led support can reinforce self-management and improve HbA1c, while lifestyle-medicine case-management roles may help coordinate education, monitoring, referrals, and follow-up [5,7,31,32]. The present negative association should therefore not be used to reduce self-management support; rather, it shows that a recent behavioral questionnaire cannot reconstruct years of cumulative exposure.

### 4.2. Strengths and Limitations

Strengths include complete item-level DSMQ data, transparent rescoring, reproducible range checks, clinically motivated alternative outcome constructions, robust standard errors, and explicit assessment of model dispersion and fit. The conclusion concerning DSMQ was consistent across all requested sensitivity analyses.

The cross-sectional, single-center design precludes temporal inference and limits external validity. Participants were evaluated in a specialized outpatient setting, and their clinical complexity and frequency of documented comorbidities may differ from those of people with T2D managed in primary care or community settings. The findings may therefore not be generalizable to other healthcare systems, geographic regions, disease-severity distributions, or linguistic and cultural contexts. In addition, the DSMQ is self-reported and may perform differently across populations with different levels of health literacy, access to diabetes education, and availability of multidisciplinary care. Recruitment was conducted at a single specialized outpatient center. Although the screening records were verified, the single-center recruitment strategy may have introduced selection bias and limits the generalizability of the findings. The DSMQ is self-reported and may be affected by recall and social-desirability bias; a single administration cannot capture behavioral trajectories.

The condition fields were verified against the available source medical records using the criteria presented in Table 1. Nevertheless, the retrospective ascertainment relied on routinely documented clinical information, and the completeness and level of diagnostic detail varied across conditions. Information on disease onset, severity and phenotype was not consistently available, which may have resulted in residual outcome misclassification. Equal weighting of heterogeneous conditions remains a limitation even after sensitivity analyses. Important potential confounders—including health literacy, diabetes distress, depression, medication adherence, cumulative blood pressure and lipid exposure, smoking dose, socioeconomic status, and access to care—were unavailable or incompletely characterized.

Finally, no a priori sample-size calculation was performed, and the fixed sample may have been insufficient to detect small effects. The models estimate cross-sectional associations and should not be interpreted as prediction models or causal estimates.

## 5. Conclusions

No evidence of an association between current DSMQ-measured self-management and the number of documented conditions was identified in this cohort. The conclusion was robust to excluding heart failure, separating microvascular and cardiovascular outcomes, using a laboratory-based renal sensitivity marker, and evaluating alternative count-model assumptions. Nevertheless, the confidence interval does not establish equivalence, and the temporal mismatch means that these findings do not imply that sustained self-management is unrelated to long-term complication risk. Prospective multicenter studies should use repeated self-management assessments, standardized diagnostic criteria, source-record adjudication, and incident outcomes.

## Figures and Tables

**Table 1:** Diagnostic criteria and source-record ascertainment procedures for the conditions included in the primary outcome.

Condition	Diagnostic Ascertainment Criterion	Source Used for Verification
Chronic kidney disease	Documented CKD diagnosis supported by eGFR < 60 mL/min/1.73 m^2^ persisting for ≥3 months and/or another documented marker of kidney damage	Serial laboratory results, nephrology/diabetes records and hospital discharge documentation
Diabetic peripheral neuropathy	Clinician-documented diabetic peripheral neuropathy based on compatible symptoms and neurological examination and/or an available confirmatory test	Neurology/diabetes consultation and hospital records
Diabetic retinopathy	Retinopathy documented following ophthalmological examination	Ophthalmology report or specialist medical record
Previous myocardial infarction	Previous myocardial infarction documented in a cardiology or hospital record and supported, where available, by ECG, biomarker, imaging or discharge documentation	Cardiology records and hospital discharge summaries
Stroke	Previous ischemic or hemorrhagic stroke documented clinically and/or by neuroimaging	Neurology records, CT/MRI reports or hospital discharge summaries
Peripheral arterial disease	Clinician-documented peripheral arterial disease supported by vascular examination, ankle–brachial index and/or vascular imaging, where available	Vascular medicine/surgery records and imaging reports
Heart failure	Clinician-documented heart failure supported by compatible symptoms or signs and objective evidence of cardiac dysfunction	Cardiology records, echocardiography reports and hospital discharge summaries

Sensitivity outcomes were prespecified for the revision: (1) a six-condition count excluding heart failure; (2) a microvascular count comprising recorded chronic kidney disease, peripheral neuropathy, and retinopathy; (3) a cardiovascular count comprising myocardial infarction, stroke, peripheral arterial disease, and heart failure; (4) a macrovascular count excluding heart failure; and (5) a seven-component count substituting eGFR < 60 mL/min/1.73 m^2^ at assessment for the recorded CKD field.

**Table 2:** Selected characteristics and recorded condition frequencies. Values are median (IQR) or *n* (%). Abbreviations: CKD, chronic kidney disease; DSMQ, Diabetes Self-Management Questionnaire; eGFR, estimated glomerular filtration rate; HbA1c, glycated hemoglobin; IQR, interquartile range; T2D, type 2 diabetes.

Characteristic	Available *n*	Value
Age, years	262	64.0 (58.0–73.0)
Female database code	261	144 (55.2%)
T2D duration, years	262	7.0 (4.0–14.0)
HbA1c, %	261	7.3 (6.5–8.7)
eGFR, mL/min/1.73 m^2^	262	80.3 (56.3–93.2)
DSMQ Sum Scale	262	5.10 (3.13–7.08)
All-condition count	262	3.0 (2.0–3.0)
Recorded CKD	262	171 (65.3%)
Recorded peripheral neuropathy	262	166 (63.4%)
Recorded retinopathy	262	41 (15.6%)
Recorded myocardial infarction	262	17 (6.5%)
Recorded stroke	262	18 (6.9%)
Recorded peripheral arterial disease	262	26 (9.9%)
Recorded heart failure	262	207 (79.0%)

**Table 3:** Adjusted association of DSMQ Sum Scale with alternative condition counts (*n* = 260). All models were adjusted for age, female database code, T2D duration, and HbA1c and used HC3 robust standard errors. Abbreviations: CI, confidence interval; CKD, chronic kidney disease; DSMQ, Diabetes Self-Management Questionnaire; eGFR, estimated glomerular filtration rate; HbA1c, glycated hemoglobin; IRR, incidence rate ratio; T2D, type 2 diabetes.

Outcome	*n*	IRR per DSMQ Point	95% CI	*p*
All seven recorded conditions	260	0.988	0.962–1.014	0.355
Six-condition count excluding heart failure	260	0.993	0.960–1.027	0.688
Microvascular count	260	0.993	0.964–1.024	0.665
Cardiovascular count including heart failure	260	0.980	0.945–1.015	0.252
Macrovascular count excluding heart failure	260	0.991	0.881–1.114	0.876
Seven-condition count substituting eGFR < 60 for recorded CKD	260	0.983	0.953–1.014	0.290

**Table 4:** Robust Poisson regression for the seven-condition count (*n* = 260). Abbreviations: CI, confidence interval; DSMQ, Diabetes Self-Management Questionnaire; HbA1c, glycated hemoglobin; IRR, incidence rate ratio; T2D, type 2 diabetes.

Variable	IRR	95% CI	*p*
DSMQ Sum Scale (per point)	0.988	0.962–1.014	0.355
Age (per year)	1.023	1.016–1.029	<0.001
Female database code	0.820	0.732–0.918	<0.001
T2D duration (per year)	1.008	1.000–1.016	0.037
HbA1c (per 1%)	0.994	0.956–1.034	0.777

The primary model showed Pearson χ^2^/df = 0.587 and deviance/df = 0.702, indicating underdispersion rather than overdispersion. The observed zero frequency was 8.5% compared with a Poisson-predicted 10.2%, the maximum predicted count was 4.17, and AIC was 859.2. In a negative binomial sensitivity model, the dispersion parameter was approximately 0 (α = 9.4 × 10^−9^), the DSMQ estimate was unchanged, and AIC increased to 861.2. The negative binomial model therefore provided no evidence of improved fit.

## Data Availability

The data supporting this analysis are available from the corresponding author upon reasonable request, subject to ethical and privacy restrictions.

## Supplementary Materials

The following supporting information can be downloaded at https://www.mdpi.com/article/10.3390/medicina62091777/s1. Table S1: Expanded participant characteristics. Values are median (IQR) or *n* (%). Abbreviations are defined in the table notes. Table S2: Corrected DSMQ Sum Scale and domain scores. Higher scores indicate more effective current self-management. Abbreviations: DSMQ, Diabetes Self-Management Questionnaire; IQR, interquartile range; SD, standard deviation. Table S3: Distribution of the seven-condition count and corrected DSMQ Sum Scale. Abbreviations: DSMQ, Diabetes Self-Management Questionnaire; IQR, interquartile range. Table S4: Corrected DSMQ Sum Scale by individual recorded condition. Mann–Whitney U tests with Benjamini–Hochberg correction. Abbreviations: DSMQ, Diabetes Self-Management Questionnaire; FDR, false discovery rate; IQR, interquartile range. Table S5: Correlations between the seven-condition count and corrected DSMQ scores. Abbreviations: DSMQ, Diabetes Self-Management Questionnaire; FDR, false discovery rate. Table S6: Logistic sensitivity analysis for the exploratory threshold of at least four conditions (*n* = 260). This threshold is not clinically validated. Abbreviations: BMI, body mass index; CI, confidence interval; DSMQ, Diabetes Self-Management Questionnaire; HbA1c, glycated hemoglobin; OR, odds ratio. Table S7: STROBE checklist for cross-sectional studies. Abbreviation: STROBE, Strengthening the Reporting of Observational Studies in Epidemiology.

## Abbreviations

The following abbreviations are used in this manuscript:

Abbreviation	Definition
AIC	Akaike information criterion
BMI	Body mass index
CI	Confidence interval
CKD	Chronic kidney disease
DSMQ	Diabetes Self-Management Questionnaire
eGFR	Estimated glomerular filtration rate
FDR	False discovery rate
HbA1c	Glycated hemoglobin
IQR	Interquartile range
IRR	Incidence rate ratio
OR	Odds ratio
SD	Standard deviation
STROBE	Strengthening the Reporting of Observational Studies in Epidemiology
T2D	Type 2 diabetes
