# Supplementary material for: Current Diabetes Self-Management and the Number of Documented Complications in Adults with Type 2 Diabetes: A Cross-Sectional Study
Source: Medicina (Kaunas). 2026 Sep 16;62(9):1777. doi: 10.3390/medicina62091777 (PMC13609716; doi:10.3390/medicina62091777)
Supplement: Supplementary file 1 [file medicina-62-01777-s001.zip › medicina-4549093-supplementary.pdf]

Table S1. Expanded participant characteristics. Values are median (IQR) or n (%). Abbreviations are defined in the table notes.

Characteristic	Available n	Value
Age, years	262	64.0 (58.0–73.0)
Female database code	261	144 (55.2%)
Rural residence	262	147 (56.1%)
Family support	262	215 (82.1%)
Reported physical activity	262	185 (70.6%)
Current smoking	262	62 (23.7%)
Alcohol consumption	262	29 (11.1%)
T2D duration, years	262	7.0 (4.0–14.0)
Hospitalizations in previous year, n	262	1.0 (1.0–2.0)
Recorded severe hyperglycemia episodes, n	262	3.0 (2.0–3.0)
Recorded severe hypoglycemia episodes, n	262	0.0 (0.0–0.0)
Weight, kg	262	84.0 (75.0–99.0)
BMI, kg/m^2 (recalculated)	262	31.0 (27.8–34.9)
Waist circumference, cm	262	108.0 (100.0–118.0)
Systolic blood pressure, mmHg	262	142.5 (130.0–158.0)
Diastolic blood pressure, mmHg	262	85.0 (78.0–92.0)
Fasting plasma glucose, mg/dL	261	140.0 (121.0–168.0)
HbA1c, %	261	7.3 (6.5–8.7)
Total cholesterol, mg/dL	262	174.0 (136.0–202.8)
Triglycerides, mg/dL	262	152.0 (99.2–256.0)
LDL cholesterol, mg/dL	262	103.0 (71.2–126.0)
HDL cholesterol, mg/dL	262	41.0 (34.0–49.0)
eGFR, mL/min/1.73 m^2	262	80.3 (56.3–93.2)
Self-monitoring of blood glucose	262	121 (46.2%)
Hospital diabetes education	262	202 (77.1%)
Individual diabetes counseling	262	61 (23.3%)
Oral antidiabetic medication	262	226 (86.3%)
Any insulin treatment	262	114 (43.5%)
Arterial hypertension	262	262 (100.0%)
Dyslipidemia	262	262 (100.0%)

Abbreviations: BMI, body mass index; eGFR, estimated glomerular filtration rate; HbA1c, glycated hemoglobin; HDL, high-density lipoprotein; IQR, interquartile range; LDL, low-density lipoprotein; T2D, type 2 diabetes.

Table S2. Corrected DSMQ Sum Scale and domain scores. Higher scores indicate more effective current self-management. Abbreviations: DSMQ, Diabetes Self-Management Questionnaire; IQR, interquartile range; SD, standard deviation.

Scale	n	Mean ± SD	Median (IQR)	Range
DSMQ Sum Scale	262	5.26 ± 2.35	5.10 (3.13–7.08)	0.63–10.00
Glucose Management	262	6.22 ± 2.35	6.00 (4.00–8.00)	1.33–10.00
Dietary Control	262	4.20 ± 3.25	4.17 (1.67–6.67)	0.00–10.00

Physical Activity	262	5.27 ± 3.40	5.56 (2.22–8.89)	0.00–10.00
Health Care Use	262	6.16 ± 2.59	6.67 (3.33–7.78)	0.00–10.00

Table S3. Distribution of the seven-condition count and corrected DSMQ Sum Scale. Abbreviations: DSMQ, Diabetes Self-Management Questionnaire; IQR, interquartile range.

**Condition count**	**Participants, n**	**%**	**DSMQ median (IQR)**
0	22	8.4	5.42 (3.80–7.34)
1	33	12.6	6.04 (3.96–7.29)
2	75	28.6	5.00 (2.71–6.56)
3	77	29.4	5.00 (2.71–7.08)
4	44	16.8	4.79 (3.65–7.19)
5	10	3.8	4.90 (3.65–6.15)
6	1	0.4	2.71 (2.71–2.71)

Table S4. Corrected DSMQ Sum Scale by individual recorded condition. Mann–Whitney U tests with Benjamini–Hochberg correction. Abbreviations: DSMQ, Diabetes Self-Management Questionnaire; FDR, false discovery rate; IQR, interquartile range.

**Condition**	**Present: n, median (IQR)**	**Absent: n, median (IQR)**	**Raw p**	**FDR p**
CKD	171: 5.21 (3.13–6.98)	91: 5.00 (3.13–7.29)	0.656	0.919
Peripheral neuropathy	166: 4.90 (2.76–7.03)	96: 5.52 (3.28–7.29)	0.286	0.919
Retinopathy	41: 4.79 (2.92–7.08)	221: 5.21 (3.13–7.29)	0.844	0.985
Myocardial infarction	17: 4.79 (3.33–6.25)	245: 5.21 (3.13–7.08)	0.995	0.995
Stroke	18: 5.21 (3.96–7.14)	244: 5.10 (3.07–7.08)	0.626	0.919
Peripheral arterial disease	26: 4.58 (2.92–5.99)	236: 5.21 (3.13–7.29)	0.476	0.919
Heart failure	207: 5.00 (2.71–7.29)	55: 5.83 (3.85–6.67)	0.309	0.919

Table S5. Correlations between the seven-condition count and corrected DSMQ scores. Abbreviations: DSMQ, Diabetes Self-Management Questionnaire; FDR, false discovery rate.

**DSMQ scale**	**Spearman ρ**	**Raw p**	**FDR-adjusted p**
DSMQ Sum Scale	−0.056	0.368	0.684
Glucose Management	−0.019	0.759	0.942
Dietary Control	−0.005	0.942	0.942
Physical Activity	−0.093	0.133	0.667
Health-Care Use	−0.051	0.411	0.684

Table S6. Logistic sensitivity analysis for the exploratory threshold of at least four conditions (n = 260). This threshold is not clinically validated. Abbreviations: BMI, body mass index; CI, confidence interval; DSMQ, Diabetes Self-Management Questionnaire; HbA1c, glycated hemoglobin; OR, odds ratio.

**Variable**	**OR**	**95% CI**	**p**
DSMQ Sum Scale (per point)	1.055	0.908–1.225	0.487
Age (per year)	1.056	1.020–1.093	0.002
Female database code	0.500	0.261–0.956	0.036
T2D duration (per year)	1.020	0.974–1.069	0.398
BMI (per kg/m^2)	1.030	0.977–1.086	0.278

HbA1c (per 1%)	1.072	0.880–1.306	0.488
Any insulin treatment	1.186	0.594–2.368	0.629
Individual counseling	1.476	0.713–3.057	0.295

Table S7. STROBE checklist for cross-sectional studies. Abbreviation: STROBE, Strengthening the Reporting of Observational Studies in Epidemiology.

Item	Topic	Recommendation	Manuscript location
1	Title and abstract	Design and balanced summary	Title; Abstract
2	Background/rationale	Scientific background and rationale	Introduction, paragraphs 1–4
3	Objectives	Specific objectives and hypotheses	Introduction, final paragraph
4	Study design	Key design elements early	Methods 2.1
5	Setting	Setting, location, and dates	Methods 2.1
6	Participants	Eligibility and selection methods	Methods 2.1; Figure 1
7	Variables	Outcomes, exposures, predictors, confounders	Methods 2.2–2.5
8	Data sources/measurement	Sources and measurement methods	Methods 2.2–2.4; Table 1
9	Bias	Efforts to address potential bias	Methods 2.2; Discussion 4.2
10	Study size	How study size was determined	Methods 2.5
11	Quantitative variables	Handling of quantitative variables	Methods 2.2–2.5
12	Statistical methods	Analyses, confounding, missing data, sensitivity	Methods 2.5
13	Participants	Numbers at each stage and flow	Results 3.1; Figure 1
14	Descriptive data	Participant characteristics and missingness	Results 3.1–3.2; Tables 2 and S1
15	Outcome data	Numbers or summary measures	Results 3.2; Tables 2 and S3
16	Main results	Unadjusted/adjusted estimates and precision	Results 3.3–3.4; Tables 3–4
17	Other analyses	Subgroups, interactions, sensitivity	Results 3.3–3.4; Tables 3 and S4–S6
18	Key results	Summary with reference to objectives	Discussion, opening paragraph
19	Limitations	Bias/imprecision and direction/magnitude	Discussion 4.2
20	Interpretation	Cautious overall interpretation	Discussion; Conclusions
21	Generalisability	External validity	Discussion 4.2
22	Funding	Source and role of funders	Funding statement
